# Identification of Decrease in TRiC Proteins as Novel Targets of Alpha-Amanitin-Derived Hepatotoxicity by Comparative Proteomic Analysis In Vitro

**DOI:** 10.3390/toxins13030197

**Published:** 2021-03-09

**Authors:** Doeun Kim, Sunjoo Kim, Ann-Yae Na, Chang Hwan Sohn, Sangkyu Lee, Hye Suk Lee

**Affiliations:** 1 BK21 FOUR Community-Based Intelligent Novel Drug Discovery Education Unit, College of Pharmacy and Research Institute of Pharmaceutical Sciences, Kyungpook National University, Daegu 41566, Korea; kdkdl1230@gmail.com (D.K.); cpblady@daum.net (A.-Y.N.); 2 BK21 Four-Sponsored Advanced Program for SmartPharma Leaders, College of Pharmacy, The Catholic University of Korea, Bucheon 14662, Korea; tjswn712@nate.com; 3 Department of Emergency Medicine, Asan Medical Center, College of Medicine, University of Ulsan, Seoul 05505, Korea; schwan97@gmail.com

**Keywords:** alpha-amanitin, comparative quantitative proteomics, TRiC, hepatotoxicity

## Abstract

Alpha-amanitin (α-AMA) is a cyclic peptide and one of the most lethal mushroom amatoxins found in *Amanita phalloides*. α-AMA is known to cause hepatotoxicity through RNA polymerase II inhibition, which acts in RNA and DNA translocation. To investigate the toxic signature of α-AMA beyond known mechanisms, we used quantitative nanoflow liquid chromatography–tandem mass spectrometry analysis coupled with tandem mass tag labeling to examine proteome dynamics in Huh-7 human hepatoma cells treated with toxic concentrations of α-AMA. Among the 1828 proteins identified, we quantified 1563 proteins, which revealed that four subunits in the T-complex protein 1-ring complex protein decreased depending on the α-AMA concentration. We conducted bioinformatics analyses of the quantified proteins to characterize the toxic signature of α-AMA in hepatoma cells. This is the first report of global changes in proteome abundance with variations in α-AMA concentration, and our findings suggest a novel molecular regulation mechanism for hepatotoxicity.

## 1. Introduction

Intake of mushrooms continues to increase owing to their significant antioxidant properties that improve health; additionally, they are an abundant source of low-calorie food diet and proteins [1]. However, as the habitat of poisonous mushrooms changes owing to climate change, there is a possibility of mixing poisonous and edible mushrooms owing to their similar appearance. Hence, poisonous mushrooms may be misused when harvesting in nature. Certain poisonous mushrooms have deadly toxins, and their consumption leads to frequent poisoning accidents worldwide [1,2]. Among mushroom toxins, amatoxins, as cyclopeptide hepatotoxin, are found in *Amanita verna*, *Amanita phalloides*, and *Lepiota helveola*. These toxins comprise alpha-amanitin (α-AMA), β-AMA, γ-AMA, amaninamide, amanin, etc. [3].

α-AMA is one of the most lethal mushroom toxins and a leading cause of morbidity, which plays a significant role in *A. phalloides* poisoning [2]. The liver is the primary organ targeted by α-AMA owing to the gastrointestinal absorption of amatoxins, and acute liver failure (ALF) is a representative consequence of its deadly toxicity [4]. Previous studies have indicated several mechanisms of α-AMA hepatotoxicity. First, α-AMA is absorbed through the intestinal epithelium, and uptake is accomplished via the OATP1B3 transporter located in the sinusoidal membrane of hepatocytes [5]. Once within the hepatocyte, α-AMA directly binds to RNA polymerase II and inhibits its activity of interfering with DNA and RNA translocation, thereby resulting in cell death [6,7,8]. Hence, as the mRNA level decreases, protein synthesis decreases, eventually leading to cell death.

Although ALF is important to α-AMA, diagnosis is difficult because the most significant symptoms of poisoning, such as nausea, vomiting, and diarrhea, appear almost 24 h after ingestion [9,10]. Several investigations regarding α-AMA hepatotoxicity mechanism and its therapeutic potential in pancreatic carcinoma have been conducted [11,12]. However, the content of global proteome analysis for the identification of markers pertaining to the diagnosis and the mechanisms of liver toxicity induced by α-AMA exposure is limited. Here, we treated Huh-7 human hepatoma cells with α-AMA at concentrations of 2, 5, and 10 μM to induce toxicity, and we analyzed protein changes via quantitative mass spectrometry analysis with tandem mass tag (TMT) labeling. Moreover, the properties of the changed proteins were identified through bioinformatics analyses such as Gene Ontology (GO), Kyoto Encyclopedia of Genes and Genomes (KEGG) pathways, InterPro, and protein networking analysis [13,14,15,16].

## 2. Results

### 2.1. Increased Toxicity in Hepatoma Cells by α-Amanitin Treatment

**Figure 1:**
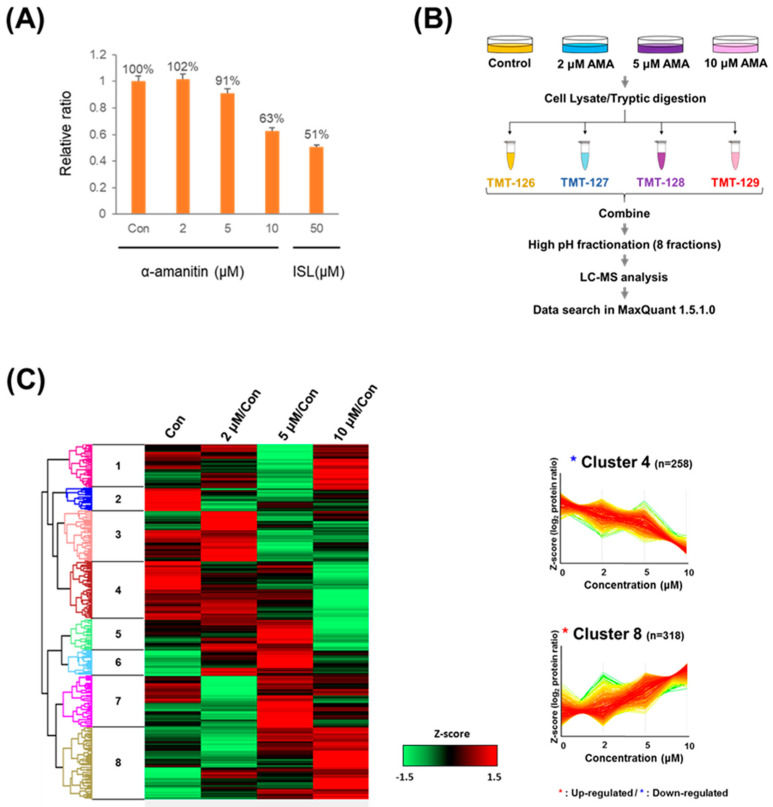
Global proteomics of α-amanitin (α-AMA) in Huh-7 human hepatoma cells. Determination of α-AMA cytotoxicity in Huh-7 cells (**A**). Sample preparation for quantitative proteome analysis (**B**). Heatmap between control (Con) and differently-treated α-AMA groups following Z-score normalization (**C**). Huh-7 cells (1 × 10^5^/mL) were seeded in a 24-well plate. Next, cells were treated with α-AMA (2, 5, and 10 μM) and were incubated for 24 h. Isoliquiritigenin (ISL) was used as a positive control, and cytotoxicity was measured at a wavelength of 450 nm. The cells were harvested and lysed to collect soluble proteins. After tryptic digestion, peptide tandem mass tag labeling was performed for quantitative proteomics.

α-AMA was treated by concentration to evaluate cytotoxicity and determine the treatment concentration of α-AMA in hepatoma cells for quantitative proteomic studies. Although no cytotoxicity was observed following treatment with 2 μM of α-AMA in our study, a previous report found that 2 μM of α-AMA facilitated hepatic damage that could cured in a human normal liver cell line [12]. In our study, treatment with 5 and 10 μM of α-AMA resulted in the death of approximately 10% and 40% of cells using Cell Counting Kit-8 (CCK-8) (Figure 1A). Although the CCK assay does not measure direct cell death, the measured CCK value indicates a significant toxic effect of α-AMA on the cells. In accordance with these results, we assigned these concentrations as curable (2 μM), LC10 (5 μM), and LC40 (10 μM) to determine the proteome changes from early-stage to lethal-stage hepatic failure.

### 2.2. Quantitative Proteomic Analysis

We used quantitative proteomic analysis (Figure 1B) to identify 1828 proteins and consequently quantified 1563 proteins filtered with a MaxQuant score of >40 and a false discovery rate of < 1% (Supplemental Table S1). Next, we divided all reported ion intensities of the treatment groups by the reporter ion intensity of a control group. We normalized these values by Z-score normalization to group proteins according to tendencies of changes in abundance. Hierarchical clustering based on Euclidean distance was used to cluster the normalized scores, and average linkage clustering was used to process the k-means. These results were visualized by heat map clustering (Figure 1C, Supplemental Table S2). Clusters 4 (n = 258) and 8 (n = 318) were identified as down- and upregulating groups, respectively, because they showed α-AMA concentration-dependent protein abundance changes. Therefore, these two clusters were analyzed using DAVID to annotate GO, InterPro, and KEGG pathway analyses, with Fisher’s exact test *p*-value of < 0.05.

### 2.3. Identification of New Targets through Bioinformatics Analysis

**Figure 2:**
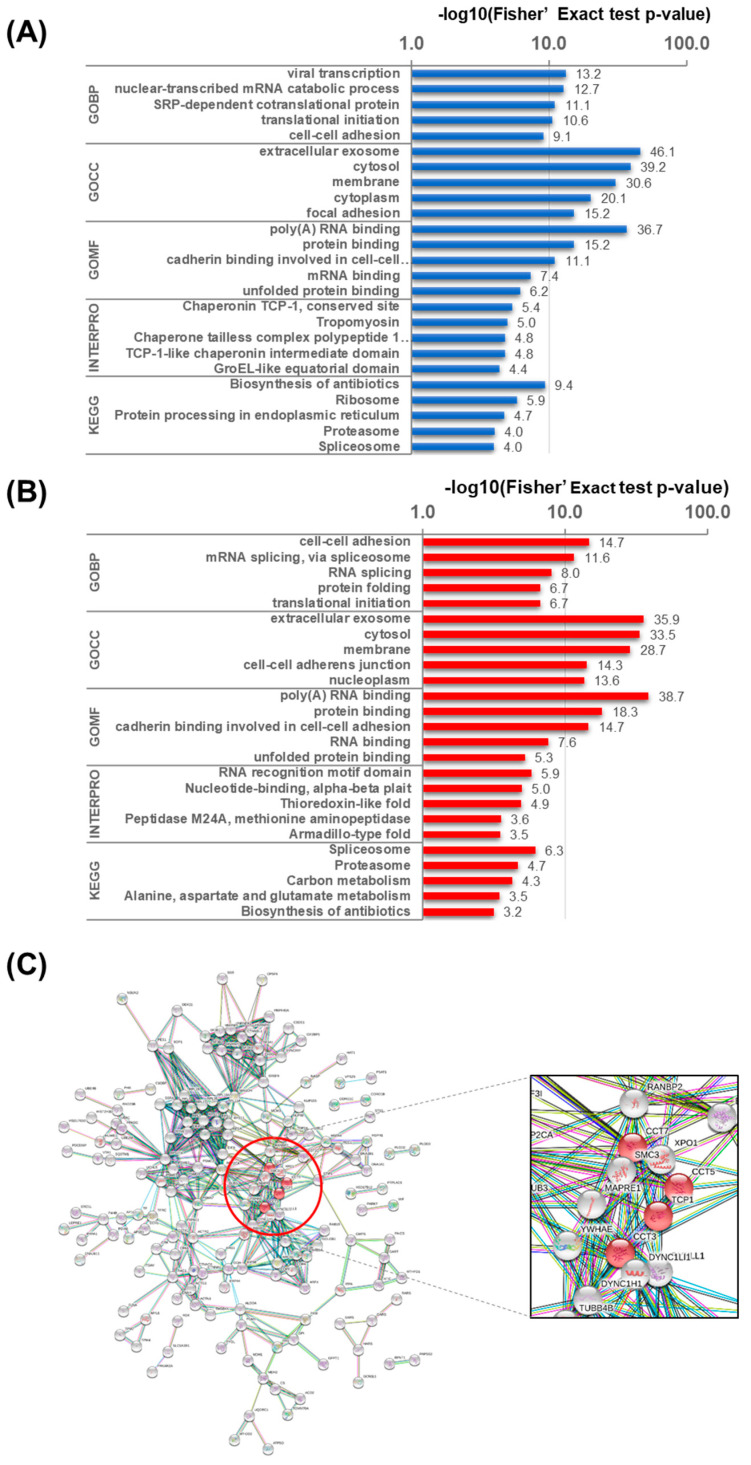
Bioinformatics results of up- and downregulated proteins. Gene Ontology (GO) enrichment, InterPro, and Kyoto Encyclopedia of Genes and Genomes (KEGG) analyses of downregulated proteins (**A**) and upregulated proteins (**B**). GOBP, Gene Ontology Biological Process; GOCC, Gene Ontology Cellular Composition; GOMF, Gene Ontology Molecular Function. Protein functional networking analysis of cluster 4 (downregulated proteins) (**C**). The confidence score was set at 0.9.

We performed GO analysis consisting of Biological Process, Cellular Composition, and Molecular Function annotations (Figure 2A,B). In the Biological Process category, cell–cell adhesion, mRNA splicing, via spliceosome, RNA splicing, protein folding, and translational initiation were upregulated (Figure 2B), whereas viral transcription, nuclear-transcribed mRNA catabolic process, nonsense-mediated decay, SRP-dependent cotranslational protein targeting to membrane, and the translational initiation process were downregulated (Figure 2A). Molecular function analysis was the same for each protein (poly(A) RNA binding, protein binding, cadherin binding involved in cell–cell adhesion, mRNA binding, and unfolded protein binding). However, RNA-binding proteins for RNA translation were upregulated, and proteins for RNA transcription were downregulated.

Furthermore, we conducted InterPro protein sequence analysis and KEGG pathway analysis to classify the role of these proteins (Figure 2A,B). The expression of RNA recognition motif domain, nucleotide-binding, alpha-beta plait, thioredoxin-like fold, peptidase M24A, methionine aminopeptidase, subfamily 2, binding site, and armadillo-type fold increased (Figure 2B), whereas that of chaperonin-containing TCP-1, tropomyosin, TCP-1-like chaperonin intermediate domain, and GroEL-like equatorial domain decreased in the InterPro analysis results (Figure 2A). In the KEGG pathway analysis, spliceosome, proteasome, carbon metabolism, alanine, aspartate and glutamate metabolism, and biosynthesis of antibiotics were upregulated (Figure 2B), whereas biosynthesis of antibiotics, ribosome, protein processing in endoplasmic reticulum, and proteasome were downregulated (Figure 2A).

To determine the protein association networks, we performed STRING protein–protein interaction prediction analysis on the up- and downregulated groups (Figure 2C). We focused on the downregulated group that had InterPro results related to protein folding. Next, we mapped four chaperonin-containing TCP1 complex (CCT) subunits (CCTα, CCTγ, CCTε, and CCTη known as CCT1, CCT3, CCT5, and CCT7, respectively) to the downregulated group, with the interaction confidence score set to 0.9 (Figure 2C).

### 2.4. Immunoblot Validation of Reduction of TRiC Proteins

**Figure 3:**
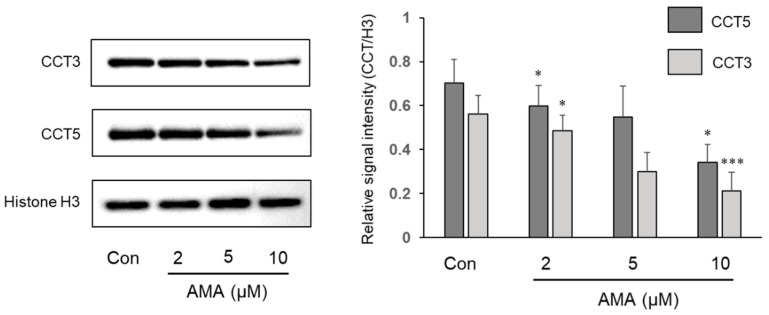
Validation of proteomic results by western blot analysis of Huh-7 lysates. Using antibodies against CCT3 and CCT5, the CCT3 and CCT5 signal intensities were calculated by dividing with the Histone H3 signal intensity which acted as a loading control. The results are presented as mean ± S.E. and were compared based on the control sample intensity. Paired t-test was used to calculate the level of statistical significance (* *p* < 0.05, *** *p* < 0.001).

To validate the proteomic results, we selected the two most decreased proteins CCT3 and CCT5 of TRiC subunits in global proteomic data to execute a western blot analysis (Figure 3). Histone H3 was used as a loading control to normalize the CCT3 and the CCT5 signal intensities. The normalized CCT3 and CCT5 signal intensities were gradually decreased in western blot analysis according to α-AMA concentrations, which represents that the western blot results matched well with the global proteomic results.

## 3. Discussion

α-AMA is a major lethal mushroom toxin; however, the subsequent poisoning symptoms appear very slowly, thereby rendering the diagnosis of mushroom toxin poisoning is very difficult. Here, we used comparative quantitative nanoflow liquid chromatography–tandem mass spectrometry analysis to demonstrate that the hepatic proteome changes after treatment with several concentrations of α-AMA. The proteome results were divided into eight clusters by unsupervised hierarchical clustering. Bioinformatics results indicated that inaccurate mRNA transcription and translation as well as levels of misfolded skeletal proteins increased.

Abundance of some T-complex protein-1 ring complex (TRiC) subunit changed, resulting in skeletal protein misfolding that could lead to hepatotoxicity occurring owing to cell death. CCT, also known as TRiC, comprises CCTα, β, γ, δ, ε, ζ, η, and θ subunits (also known as CCT1–CCT8) assembled into a double-ring structure [17,18,19]. Eukaryotic chaperonins are involved in polypeptide folding by ATP binding and hydrolysis, particularly in tubulin, actin, and skeletal structure-related proteins [20,21,22]. The dysfunctions of TRiC/CCT were reported in several diseases related to cancer and neurodegenerative diseases, such as breast cancer, colorectal cancer, hepatocellular cancer, Huntington’s, Alzheimer’s, and Parkinson’s disease [23,24,25,26,27,28,29]. Moreover, there are several reports pertaining to biological processes, including cell death, autophagy, cell polarity, morphogenesis, and cancer signaling [30,31,32,33,34]. Four CCT subunits were α-AMA concentration-dependently decreased in our results, indicating that cellular structures were unstable owing to declining levels of skeletal proteins. 

Our findings suggest that both protein dysfunction and skeletal protein misfolding trigger unstable conditions in liver cells. Because the current research was conducted on human hepatocellular carcinoma cell line (Huh-7), it can reflect the hepatotoxicity mechanism that may appear clinically. Although it should be verified as a new treatment target through additional in vivo systems or validation in a clinical environment, we believe that the value is high as a new clue to identify novel therapeutic targets to alleviate α-AMA-derived hepatotoxicity. Therefore, we expect that the hepatotoxicity mechanism of α-AMA caused by cell death stems from the failure of skeletal protein folding and inaccurate protein synthesis.

## 4. Materials and Methods

### 4.1. Cytotoxicity of α-AMA in Hepatoma Cells

The cytotoxicity of α-AMA was validated using CCK-8 reagent (Dojindo Molecular Technologies, Kumamoto, Japan) to determine the inhibition concentration of α-AMA prior to comparative proteomic analysis in Huh-7 cells. CCK-8 assay is a sensitive colorimetric method used for the determination of cell viability in cell proliferation and death. The amount of the formazan dye generated by dehydrogenases in cells is directly proportional to the number of living cells. The Huh-7 cells were cultured in Dulbecco’s Modified Eagle Medium (Hyclone Laboratories Inc., Erie, PA, USA) supplemented with 10% fetal bovine serum (Hyclone Laboratories Inc., Erie, PA, USA) and 1% penicillin-streptomycin (Gibco, Waltham, MA, USA) to a concentration of 1 × 10^5^/mL and were incubated for 24 h. Next, the cell medium was removed using a suction pump and was washed with 1× phosphate-buffered saline (Gibco, Waltham, MA, USA). Next, the cell medium was replaced with fresh cell media containing 1% penicillin-streptomycin and α-AMA (Tocris Bioscience, Bristol, UK) at a concentration of 2, 5, and 10 μM, respectively, and was then incubated for 24 h. Simultaneously, 50 μM isoliquiritigenin treatment was used as a positive control to induce cytotoxicity [35]. Thereafter, the cell medium was removed and fresh cell media mixed with cytotoxicity-checking reagent CCK-8 were added. The absorbance was measured at 450 nm with a spectrophotometer.

### 4.2. Sample Preparation for Quantitative Proteomics Analysis

After checking for cytotoxicity, Huh-7 cells were incubated in 100 mm Ø dishes under the same conditions as described above. Consequently, the α-AMA-treated Huh-7 cells were harvested and were directly added to 500 μL of 8 M urea (Sigma-Aldrich, St. Louis, MO, USA) in 100 mM Tris (VWR International, Radnor, PA, USA) containing protease inhibitors (Thermo Fisher Scientific, Waltham, MA, USA). The collected cells were sonicated for 1 min (output 30%, 5-s on and off intervals) and were centrifuged at R.T at 16,000× *g* for 10 min to separate the soluble proteins from the cell debris. The upper fraction was removed and placed in new sample tubes, and the protein concentration was determined using a BCA kit (Thermo Fisher Scientific). Biologically duplicated samples were harvested, and proteins were extracted from each treatment group.

We placed 100 μg of the protein samples in new sample tubes and added 5 mM of dithiothreitol (Sigma-Aldrich) for cysteine residue reduction at 56 °C for 30 min followed by treatment with 15 mM iodoacetamide (Sigma-Aldrich) for cysteine residue alkylation in the dark for 30 min. Next, a two-fold volume dilution was performed for trypsin digestion. Following a pH check, trypsin (2 μg) was directly added to the sample and left to digest for 18 h at 37 °C. Then, 1% trifluoroacetic acid (Sigma-Aldrich) was added to complete the digestion step. The peptides were dried in speed vac dryer at a low temperature and were directly dissolved in 50 mM tetraethylammonium bromide (Sigma-Aldrich) for 4-plex TMT reagent labeling (Thermo Fisher Scientific). After checking the peptide concentration using a Pierce™ Quantitative Colorimetric Peptide Assay Kit (Thermo Fisher Scientific), equal amounts of peptides from each group were labeled and placed in one sample tube. The pooled peptide samples were fractionated using a Pierce™ High pH Reversed-Phase Peptide Fractionation Kit (Thermo Fisher Scientific).

### 4.3. Quantitative Proteomics Analysis

The fractionated samples were dissolved in 10 μL of solution A (2% acetonitrile in 0.1% formic acid), and 500 ng of each fraction were loaded onto a nanoLC 1D plus system (Eksigent, Walpole, MA, USA) consisting of an in-house C12 resin (4 µm Proteo 90 Å; Phenomenex Inc., Torrance, CA, USA) and a capillary column (ID 75 µm, OD 150 µm; Molex, Lisle, IL, USA). Elution was conducted using a gradient liquid chromatography method (0–25% acetonitrile for 90 min) and was analyzed with an LTQ-Orbitrap Velos mass spectrometer (Thermo Fisher Scientific) in positive ion mode at the Mass Spectrometry Convergence Research Center. The *m*/*z* data collection range was set at 300–1800 *m*/*z*, and a higher-energy collisional dissociation collision mode was used for fragmentation. The quantitative mass spectrometry analyses were technically duplicated for each of the pooled peptide samples (n = 4).

All mass spectra data were input to MaxQuant 1.5.1.0 to obtain bioinformatics information, and the human proteome database (updated 12/13/2018) was downloaded from Uniprot (https://www.uniprot.org/proteomes/UP000005640, accessed on 8 March 2021). GO, InterPro, and KEGG pathways were analyzed using DAVID Functional Annotation Bioinformatics Microarray Analysis (https://david.ncifcrf.gov/, accessed on 8 March 2021). Perseus 1.6.0.7 (Max-Planck-Institute of Biochemistry, Planegg, Germany) was used for clustering protein groups depending on the protein regulation patterns. The STRING analytical tool (https://string-db.org/, accessed on 8 March 2021) was used to search specific protein networks according to regulation differences resulting from the α-AMA treatments at different concentrations.

## Data Availability

Data are available via ProteomeXchange with identifier PXD018702.

## Supplementary Materials

The following are available online at https://www.mdpi.com/2072-6651/13/3/197/s1, Table S1: All quantified protein list; Table S2: (A–H) Protein list for Cluster (1–8).
